# Preparation of Infrared Anti-Reflection Surfaces Based on Microcone Structures of Silicon Carbide

**DOI:** 10.3390/ma18174054

**Published:** 2025-08-29

**Authors:** Ruirui Li, Xiaozheng Ji, Sijia Chang, Haoyu Tian, Zihong Zhao, Chengqun Chu

**Affiliations:** State Key Laboratory of Extreme Environment Optoelectronic Dynamic Measurement Technology and Instrument, North University of China, Taiyuan 030051, China; xiaozhengji2024@163.com (X.J.); csj15035900484@163.com (S.C.); 18574427215@163.com (H.T.); zhaohh0436@163.com (Z.Z.); chuchengqun@nuc.edu.cn (C.C.)

**Keywords:** SiC, microcone, anti-reflection, infrared optics, RIE

## Abstract

Silicon carbide (SiC) has become the material of choice for precision optical systems due to its exceptional optical characteristics. However, conventional anti-reflection strategies for SiC components predominantly utilize deposited thin-film coatings, which are frequently compromised by insufficient environmental robustness and long-term stability concerns. To overcome these limitations, direct nanostructuring of SiC substrates has emerged as a promising alternative solution. This work introduces an innovative graded-index microcone array design fabricated on SiC substrates, achieving superior broadband anti-reflection performance. Our two-step fabrication methodology comprises plasma-induced formation of tunable nanofiber etch masks through controlled argon bombardment parameters, followed by precision reactive ion etching (RIE) for microcone array formation. By systematically varying plasma exposure duration, we demonstrate precise control over nanofiber mask morphology, which in turn enables the fabrication of height-optimized SiC microcone arrays. The resulting structures exhibit exceptional optical performance, achieving an ultra-low average reflectivity of 2.25% across the spectral range of 2.5–8 μm. This breakthrough fabrication technique not only extends the available toolbox for SiC micro/nanofabrication but also provides a robust platform for next-generation optical applications. Unlike conventional thin-film approaches, our nanostructuring method preserves the intrinsic mechanical and environmental durability of the SiC substrate while delivering a favorable optical performance.

## 1. Introduction

Silicon carbide (SiC) is a third-generation semiconductor material that exhibits superior performance compared to traditional silicon in multiple aspects. Owing to its high-temperature resistance, high thermal conductivity, wide bandgap, and excellent chemical stability, SiC is widely used in light-emitting diodes [1], power devices [2], and high-temperature sensors [3]. Moreover, due to its broad optical transmission range and high refractive index, SiC is also used in optical systems. For instance, it can be used as a material for mirrors [4], optical waveguides [5], and metal lenses [6]. At a wavelength of 1550 nm, SiC has a refractive index of approximately 2.6, offering a significant advantage over traditional optical waveguide materials such as glass (refractive index ~1.5). This high refractive index enables tighter confinement of optical signals, thus reducing modal loss. However, this also leads to higher optical reflection losses [7]. Therefore, research on anti-reflection technologies for SiC surfaces is critical to mitigate reflection losses in optical systems.

Currently, there are two primary technological approaches for achieving an anti-reflection surface: optical thin-film deposition and surface micro/nanostructure fabrication. For thin-film deposition, researchers have developed various material systems, including conventional fluorides [8], sulfides [9], and oxides [10], as well as novel materials such as germanium carbide films [11], nanoporous structures [12], and noble metal nanofilms. Numerous works have demonstrated that precisely designed multilayer films can significantly enhance transmittance in target wavelength ranges for substrates like sapphire, ZnS, and glass. For instance, double-sided coated sapphire achieves 96.7% visible light transmittance [13], ZnS-based films exhibit >80% average transmittance in the 400–1000 nm range [14], and coated K9 glass reaches 98.55% average transmittance across dual bands of 0.3–2.4 μm [15]. Particularly noteworthy is the dual-band (3–5 μm and 8–12 μm) anti-reflection coating on Ge substrates, which boosts average transmittance to 94% [16] and has been successfully applied in optical windows for high-speed aircraft [17]. However, thin-film technology faces a critical thermal stability limitation—when operating temperatures exceed 300 °C, the coefficient of thermal expansion mismatch between films and substrates leads to interfacial delamination, ultimately causing optical component failure.

To overcome this challenge, surface micro/nanostructures have emerged as an alternative anti-reflection surface solution. By creating graded refractive index profiles through surface morphologies (e.g., conical arrays and nanopillars), this approach eliminates the risk of film delamination while preserving the intrinsic thermal stability of the substrate. Common preparation methods for surface micro/nanostructures include electron beam lithography, focused ion beam etching, femtosecond laser etching, metal-assisted chemical etching, and nanosphere etching technology [18,19]. Wang et al. employed a one-step selective direct current plasma etching technology to fabricate large-area well-aligned nanocone arrays on SiC wafers [20]. Chiew et al. employed vapor–liquid–solid (VLS) technology to fabricate SiC nanowires and nanocones using a mixture of oil palm fibers and rice husk ash [21]. Liu et al. formed crystalline SiC nanocones via the VLS process using iron nanoparticles as catalysts [22]. Ma et al. developed an Al_2_O_3_-Si composite micro/nanostructure on silicon using self-assembled mask etching and atomic layer deposition, achieving <3.5% average reflectance in the mid-infrared band [23]. Chen et al. employed flexible ultraviolet nanoimprint lithography to fabricate high-quality subwavelength structures on convex germanium surfaces, improving average transmittance from 65.81% to 78.68% for broadband mid-infrared anti-reflection [24]. Zhao et al. fabricated pore-like subwavelength surface nanostructures on sapphire and obtained a 96.8% average transmittance for double-sided structures in the visible spectrum [25]. While current research predominantly focuses on conventional optical materials like Si, SiO_2_, and sapphire [26,27,28,29], studies on wide-bandgap semiconductor materials such as SiC—with its exceptionally high-temperature resistance—remain notably limited. Given the irreplaceable role of SiC in extreme environments (e.g., hypersonic vehicle radomes and high-temperature sensor windows), developing efficient anti-reflection surface technologies for SiC has significant engineering value. Future efforts should prioritize optimizing micro/nanofabrication techniques for SiC to expand its applications under harsh operating conditions.

Here, we propose a microcone structure based on a refractive index gradient change with excellent anti-reflection performance and high stability. Through finite-difference time-domain (FDTD) simulations, we systematically investigated the influence of key microcone structural parameters on reflectivity and optimized the optimal configuration. The etching mask was prepared by parallel processing of nanofibers based on the plasma repolymerization effect, and the microcone structure was etched on the silicon carbide substrate by reactive ion etching (RIE). The reflectance of SiC substrates with different microcone structures and a blank silicon carbide substrate was tested using Fourier-transform infrared spectroscopy (FTIR). The microcone structures constructed on SiC substrate provide a simple and practical method for the anti-reflection of optical devices and optical systems. Moreover, the infrared anti-reflection surface is expected to be applied in fields such as thermal cameras, infrared lasers, remote sensing imaging, and spectral analysis in the future, which will greatly improve the performance of these devices.

## 2. Experimental Section

### 2.1. Materials

4-inch 4H−SiC substrates (crystal orientation of (0001), N-type doping, 500 μm thick) were purchased from Beijing Tianke Heda Semiconductor Co., Ltd., Beijing, China. Polyimide (PI) photoresist was purchased from Suzhou Research Materials Micro-Nano Technology Co., Ltd., Suzhou, China.

### 2.2. Preparation of Nanofibers

A PI layer (e.g., 5 μm thick) was spin-coated onto the SiC substrate. The etching mask, namely the nanofiber mask, was fabricated by sequentially bombarding the PI photoresist with O_2_ and Ar plasma. During bombardment, the radio frequency power in plasma etching was maintained at 220 W, and the cavity pressure was maintained at 5 Pa. The flow rates of O_2_ and Ar were 130 and 100 sccm. In this experiment, keeping the time of O_2_ plasma bombardment on the PI photoresist unchanged at 20 min, nanofibers with different sizes were prepared by varying the Ar plasma bombardment time (5, 10, 20, and 30 min).

### 2.3. Preparation of SiC Microcones

Using the nanofiber forest structure as a mask, a nanofiber/microcone bilayer structure based on SiC was formed using the RIE method. In this experiment, mixed gases of SF_6_, O_2_, and Ar with gas flow rates of 60 sccm, 60 sccm, and 20 sccm were introduced. The cavity pressure was set to 4 Pa, and the cavity power was 200 W. SiC microcones with different morphologies were achieved by adjusting the size of the nanofiber mask and the RIE etching time. Finally, a buffer oxide etching (BOE) solution was used to remove the residual nanofiber structure mask on the upper layer, and SiC microcone structures were obtained. Nanofibers and SiC microcones were prepared using RIE-10NR (SAMCO International, Kyoto, Japan).

### 2.4. Characterization and Testing

The morphological structures of both nanofibers and microcones were characterized using a Scanning Electron Microscope (Gemini SEM 500, Zeiss Oberkochen, Germany). FTIR (Nicolet 6700, Thermo Fisher Scientific Inc., Madison, WI, USA) was used to measure the reflectance spectra of the SiC microcone structure.

## 3. Results and Discussion

### 3.1. Preparation of SiC Microcones

**Figure 1:**
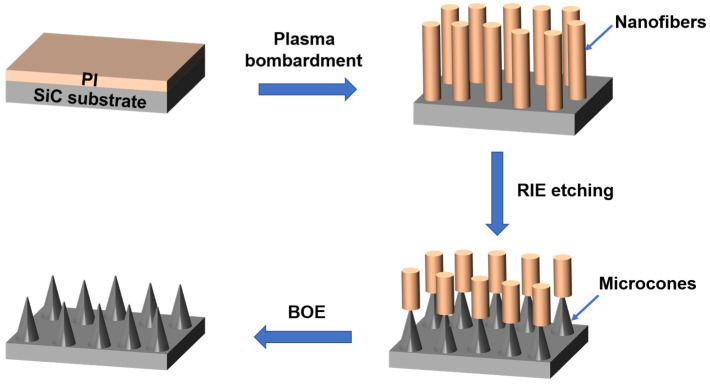
Fabrication process of SiC microcone structure.

Figure 1 illustrates the fabrication process of microcone forest structures on a SiC substrate. First, the SiC substrate is cleaned using the standard RCA cleaning process. A layer of PI photoresist is spin-coated onto the substrate, followed by sequential bombardment with O_2_ and Ar plasmas to form nanofibers. For the formation of nanofibers, in the initial stage of oxygen plasma treatment of the PI layer, the outermost layer of PI decomposes into imide monomers and other low-molecular-weight compounds. As the plasma treatment proceeds, most of these compounds are removed, leaving only imide monomers on the surface of the PI layer. Subsequently, during the high-energy dissociation stage, these monomers are fragmented into ions, free electrons, neutral particles, and reaction fragments. Afterwards, these particles and fragments recombine to form a new type of plasma polymer (also known as nano-residues), which begins to present in the form of cross-linked structures. With the continuous progress of oxygen plasma treatment, the size of the PI layer decreases, continuously supplying imide monomers at a certain flow rate. At the same time, more nano-residues are synthesized and grafted onto the previously generated cotton-like structures, thereby forming vertical nanowires. Eventually, after the entire PI layer is removed, a forest of nanowires is obtained. These nanowires have extremely small diameters and heights that are slightly smaller than the thickness of the original PI layer. Moreover, they are still insufficient to serve as an etching mask; therefore, Ar plasma needs to be introduced further. During the Ar plasma treatment, the surface layers around the nanowires will be ashed and dissociated, and then recombined to form nano-residues. Due to electrostatic attraction, these nano-residues are redeposited onto the newly formed nanowires, and the nanogaps between the nanowires are gradually filled, eventually forming robust nanofibers. Compared with the previous nanowires, the nanofibers formed at this point have a reduced height but an increased diameter per fiber, thus being able to serve as an etching mask.

Subsequently, these nanofibers serve as an etching mask for subsequent RIE, resulting in a composite structure of nanofibers and SiC microcones. The nanofibers are then removed using BOE solution, ultimately yielding the microcone structures on the SiC substrate. Since the diameter and height of the nanofiber mask determine the dimensions of the SiC microcones, the size of the microcones can be controlled by adjusting the mask parameters. The nanofiber dimensions can be tuned by varying the bombardment time of O_2_ and Ar plasmas. Additionally, the size of the SiC microcones can be precisely regulated by adjusting the etching duration. Compared with previously reported methods, such as electron beam lithography, focused ion beam etching, femtosecond laser etching, metal-assisted chemical etching, and nanosphere etching technology, this nanofiber mask fabrication method features simple operation, high speed, low cost, and high throughput. It is suitable for mass production in semiconductor manufacturing and provides a new approach for processing anti-reflection structures on SiC substrates.

### 3.2. Reflectivity Simulation Results of SiC Microcone Structure

**Figure 2:**
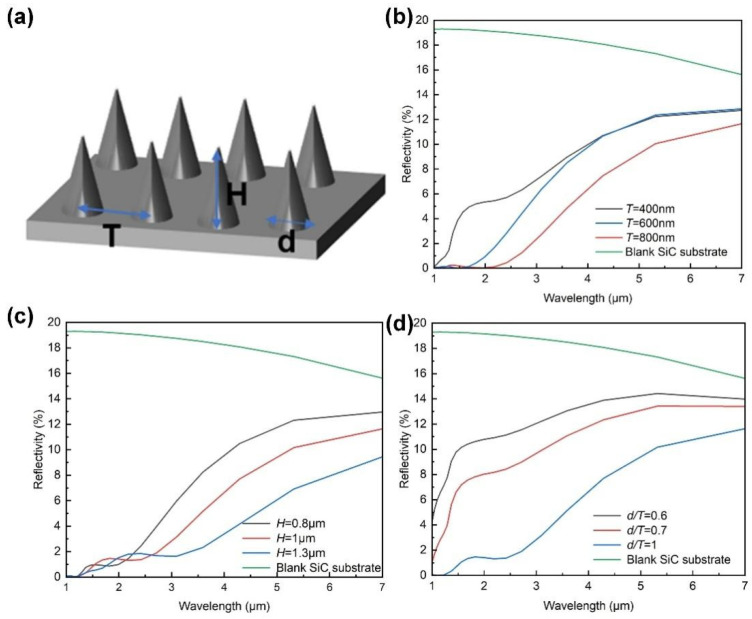
(**a**) Schematic of the physical parameters of the microcone array. Calculated spectral reflectance of SiC microcone anti-reflective structures with different (**b**) periods, (**c**) heights, and (**d**) fill factors. The optimal period, height, and fill factor are determined to be 800 nm, 1.3 μm, and 1, respectively.

The anti-reflection characteristics of SiC microcone structures were investigated through numerical simulations using the model depicted in Figure 2a. In this model, T represents the center-to-center spacing between adjacent microcones, d denotes the base diameter of a single microcone, H is the vertical height of the microcone, and the base fill factor is defined as d/T. The FDTD method was employed to simulate the reflectance of microcone structures with varying dimensions by systematically adjusting T, H, and d/T to identify the optimal structural parameters for minimal reflection. During the calculation process, the simulation domain is specifically set according to the structure being simulated. For example, if the simulation period is T, the range in the x and y directions is set to T, while the z direction takes the substrate thickness into account. A plane light source is positioned perpendicular to the base of the microcone, directed downward along the negative z-axis, and confined within the simulation domain. Regarding mesh settings, since the simulated structure is relatively small, high mesh precision is required. However, excessively high precision results in a substantial computational load. Therefore, we set the precision to 6, with a minimum mesh step size of 0.25 nm. For boundary conditions, periodic boundary conditions are applied in the x and y directions to ensure the final structure appears as a periodic array in these directions while significantly reducing simulation time. In the z direction, perfectly matched layer boundary conditions are used. Reflectance was calculated across the 1–7 μm wavelength range. To isolate the influence of the period, the base fill factor (d/T = 1) and height (H = 1000 nm) were fixed, while T was varied (400, 600, and 800 nm). As shown in Figure 2b, when T is 600 nm or 800 nm, the reflectance approaches zero in the 1–2 μm range. This phenomenon arises because when the structural period is comparable to the incident wavelength, surface plasmon resonance (SPR) and diffraction effects are excited, which confines the energy near the surface. In the 2–4 μm range, reflectance decreases with increasing microcone period. For wavelengths between 4 and 7 μm, structures with 400 and 600 nm of T exhibit similar reflectance, while microcones with 800 nm of T demonstrate the lowest reflectance. Therefore, the optimal period was determined to be 800 nm.

After determining the optimal period, the microcone period was fixed at 800 nm with a constant fill factor (d/T = 1), and the height was varied (800, 1000, and 1300 nm). Reflectance spectra are presented in Figure 2c. In the 1–3 μm short-wavelength region, all microcone heights exhibit reflectance dips, which are attributed to resonant effects when H is close to λ/2. Overall, the reflectance decreased with increasing microcone height, which can be attributed to three factors: first, the greater height enhances the thickness of the graded refractive index distribution, enabling smoother optical transitions and reducing interface reflection. Second, taller microcones strengthen the graded-index effect. Third, increased height improves light trapping through multiple internal reflections and scattering, redirecting, or absorbing incident energy. Considering the practical fabrication constraints, we selected an optimal height of approximately 1.3 μm. With the period (T = 800 nm) and height (H = 1000 nm) fixed, d/T was varied (0.6, 0.7, 1) to evaluate its impact on reflectance (Figure 2d). The results indicate that higher fill factors lead to lower reflectance. At low fill factors (e.g., 0.6), the small base diameter results in abrupt refractive-index transitions, hindering light localization and increasing reflection. As the fill factor increases, the base diameter enlarges, enhancing the graded-index effect and improving light confinement within the microcone structure. When the fill factor approaches 1, the base diameter nearly matches the period length, significantly reducing reflectance.

### 3.3. Characterization of Nanofiber Structure

**Figure 3:**
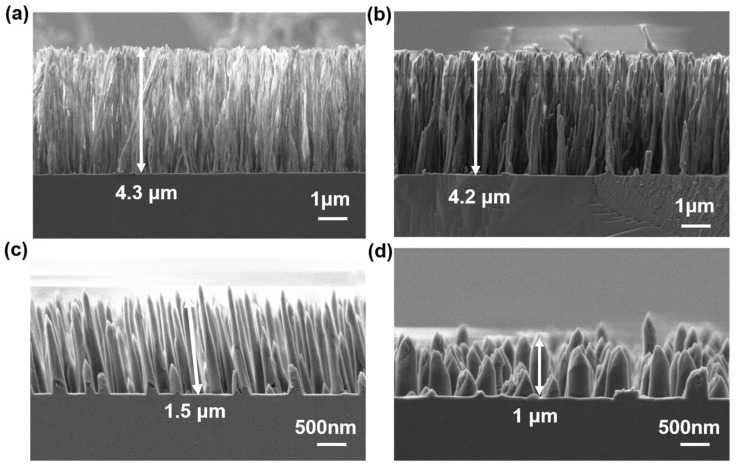
Nanofiber structures formed by Ar plasma treatment of PI photoresist for (**a**) 5 min, (**b**) 10 min, (**c**) 20 min, (**d**) 30 min. The heights of the formed nanofibers are ~4.3 μm, ~4.2 μm, ~1.5 μm, and ~1 μm.

The preparation of an etching mask is required to form SiC microcones. As mentioned above, after spin-coating photoresist on SiC, the photoresist layer is bombarded sequentially with O_2_ plasma and Ar plasma to form a nanofiber mask. The size of the nanofibers can be controlled by adjusting the duration of Ar plasma bombardment. In this experiment, the O_2_ plasma treatment time was fixed at 20 min, and by varying the Ar plasma treatment time, nanofiber forest structures with different morphological characteristics were successfully prepared, as shown in Figure 3. Figure 3a displays the nanofiber structure after 20 min of O_2_ plasma treatment and 5 min of Ar plasma treatment. It can be observed that the diameter of individual nanofibers is relatively small, with a height of approximately 4.3 μm. However, due to their slender nature, these nanofibers cannot effectively protect the underlying structure during etching, making them unsuitable as masks. Therefore, we increased the Ar plasma bombardment time. During this process, multiple nanofibers aggregate to form thicker and more robust nanofibers. Figure 3b–d show the nanofiber structures after Ar plasma bombardment for 10, 20, and 30 min. It is evident that as the argon plasma bombardment time increases, significant material accumulation and thickening occur at the base of the nanofibers. This process is accompanied by three notable structural evolution characteristics: first, the density of the nanofibers decreases; second, the diameter of individual fibers increases; and third, the spacing between nanofibers expands. After argon plasma bombardment, these fiber structures exhibit excellent mechanical strength, fully meeting the requirements for a nanomask in dry etching processes. It is particularly noteworthy that when the argon plasma bombardment time reaches 30 min, the height of the nanofibers significantly decreases. This is due to excessive bombardment, leading to the erosion of the nanofiber tips. However, this erosion does not affect the subsequent etching process.

**Figure 4:**
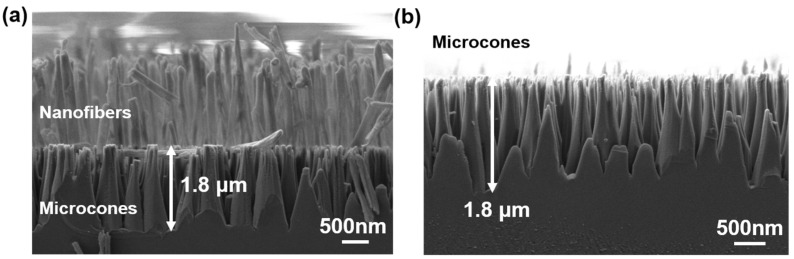
(**a**) Nanofiber/microcone double-layer structure. (**b**) SiC microcone structures after removing nanofibers with BOE. The prepared SiC microcones have a generally consistent height (~1.8 μm).

After preparing the nanofibers, they were used as a mask to etch the underlying SiC. Figure 4a shows the nanofiber/microcone bilayer structure formed after etching the SiC. During etching, the upright nanofibers shielded the silicon carbide directly beneath them, while the exposed regions were removed, resulting in the formation of individual SiC microcones. The fractured nanofibers visible in the image were caused by sample preparation during morphological observation. Figure 4b shows the SiC microcones after removing the nanofibers using the BOE solution. It can be observed that the etching process successfully produced freestanding SiC microcones with a certain height. These microcones exhibited a tapered morphology, with narrower tips and wider bases, due to the longer etching duration at the top compared to that at the bottom. This gradual size transition also contributes to improved anti-reflection properties. Throughout the entire process, the fabrication of nanofibers and SiC microcone structures both rely on highly controllable RIE parameters (e.g., precisely regulated gas composition, pressure, and power). Such strict process control is a prerequisite for the reproducible fabrication of nanostructures in semiconductor manufacturing. Therefore, maintaining the consistency of these parameters can ensure the reproducibility of key morphological features of the fabricated structures (such as microcone height and tip sharpness). Before preparing the nanofiber mask, we use spin-coating to apply the PI layer, a method capable of forming uniform films on 4-inch, 8-inch, and even 12-inch substrates. Subsequently, through RIE technology that ensures uniform energy distribution on the substrate surface, the formed nanofiber masks have uniform height, and the finally etched SiC microcones also exhibit consistent height. In other words, based on the inherent uniformity of mask deposition and plasma etching processes, large-area uniformity and reproducibility can be achieved during the fabrication of this nanostructure. These characteristics of fabrication reproducibility and uniformity provide a foundation for extending this method to production and practical applications.

### 3.4. Characterization of SiC Microcone Structure

**Figure 5:**
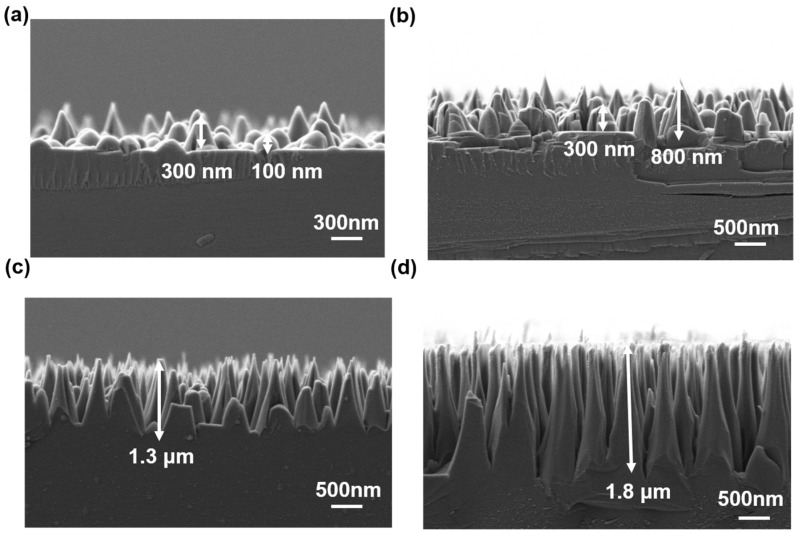
SiC microcone structures obtained by RIE etching for 10 min using nanofiber structures obtained by Ar plasma treatment of PI photoresist for (**a**) 5 min, (**b**) 10 min, (**c**) 20 min, and (**d**) 30 min as masks. The heights of the prepared SiC microcones are ~100–300 nm, ~300–800 nm, ~1.3 μm, and ~1.8 μm.

To achieve an anti-reflective surface, we fabricated microcone structures on SiC substrates using nanofibers as etching masks through the RIE process. By adjusting the morphology of the nanofibers and the RIE etching time, we were able to control the morphological characteristics of SiC microcones. We employed nanofiber masks with different structural dimensions for SiC etching, preparing masks with Ar plasma bombardment times of 5, 10, 20, and 30 min. Using these four types of masks, we performed RIE etching on SiC for 10 min under the same conditions, obtaining different silicon carbide microcone structures as shown in Figure 5. It can be observed that all four types of masks successfully formed microcone structures on the SiC substrates. Figure 5a shows the SiC microcone structure obtained using nanofibers formed by 5-min Ar plasma bombardment as the etching mask. The height of these microcones ranges from 100 to 300 nm, with poorly defined conical shapes, mostly appearing as island-like structures. This morphology results from the poor robustness of the nanofiber mask, where some nanofibers aggregate or degrade during etching, failing to fully protect the underlying SiC. Consequently, only the top surface of the SiC was etched slightly. Figure 5b displays the microcone structures obtained using nanofibers formed by 10-min Ar plasma bombardment as the mask. The measured height of these microcones ranges from 300 to 800 nm, with increased density and relatively large base diameters but lower heights. Figure 5c,d present the microcone structures obtained using nanofibers formed by 20-min and 30-min Ar plasma bombardment as masks, respectively. As the Ar plasma treatment time increases, the base diameter of the microcones gradually decreases, while both the height and aspect ratio increase accordingly. Notably, the microcones formed by 20-min and 30-min Ar plasma bombardment nanofiber masks reach heights of 1.3 μm and 1.8 μm, respectively. Meanwhile, they exhibit relatively uniform heights. This is because the highly robust nanofibers provide sufficient protection to the underlying SiC, allowing the formation of well-defined, distinct microcone structures during etching. These results demonstrate that the morphology of the microcones can be effectively controlled by adjusting the dimensions of the nanofiber masks. The morphologies of the microcones obtained using different nanofiber masks are summarized in Table 1.

**Figure 6:**
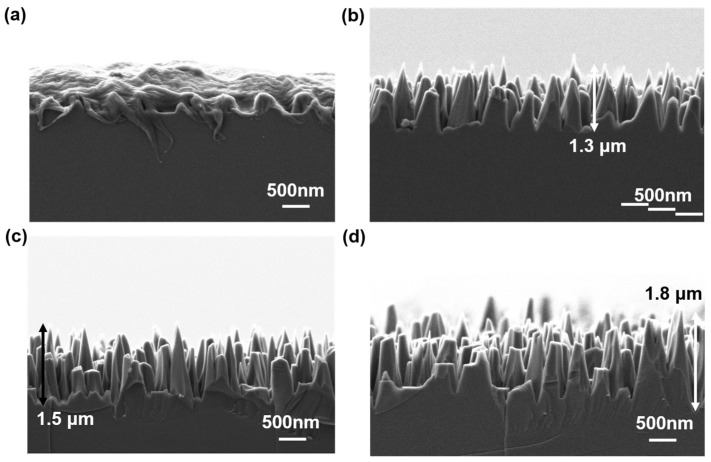
Silicon carbide microcone structure formed by etching time of (**a**) 5 min, (**b**) 10 min, (**c**) 15 min, (**d**) 20 min. The heights of the latter three microcone structures are ~1.3 μm, ~1.5 μm, and ~1.8 μm.

After determining the nanofiber mask, we investigated different morphologies of SiC microcones to achieve optimal anti-reflection performance. We systematically explored microcones fabricated with varying RIE etching times. Using nanofibers obtained through 20 min of O_2_ plasma treatment and 20 min of Ar plasma treatment as masks, we prepared microcone structures with distinct morphologies by employing different etching durations (5, 10, 15, and 20 min), as shown in Figure 6. The results demonstrate that the RIE etching time directly influences the height of microcone structures. At an etching duration of 5 min, the resulting morphology exhibited interconnected island-like structures rather than distinct conical nanostructures, with a limited height of approximately 500 nm, which proved inadequate for effective anti-reflection performance. Increasing the etching time to 10 min produced clearly defined microcones with relatively sharp tips and an approximate height of 1.3 μm. Further extending the etching time to 15 min increased the microcone height to around 1.5 μm, but the sharpness of the tips diminished, indicating that prolonged etching gradually affected the apex of the structures. At an etching time of 20 min, although the microcones reached a height of approximately 1.8 μm, most of their tips were truncated, and their aspect ratio increased accordingly. The morphologies of the obtained microcones using different etching durations are summarized in Table 2. These findings indicate that while longer etching times generally enhance microcone height and sharpen their profiles, excessive etching can blunt the tips, potentially weakening the graded refractive index effect when light interacts with the structure.

### 3.5. Antireflective Performance of SiC Microcone Structure

Regarding the anti-reflective principle of microcones, the fabricated microcone structures exhibit significant surface roughness, which is essentially determined by the geometric parameters of the cones (such as height, base width, tilt angle, distribution density, etc.). When light is incident on the surface of the microcones, multiple reflections and scattering occur on the sidewalls of the cones. The higher the roughness (e.g., the cones are denser or sharper), the more significant the multiple scattering effect, which redirects the reflected light back to the incident direction or scatters it randomly, thereby reducing the intensity of the specular reflection. In addition, the core anti-reflective mechanism of the microcone structure lies in the optimization of impedance matching through a gradient refractive index. Specifically, the densely arranged microcones can be regarded as a layer of equivalent medium at the subwavelength scale, resulting in a quasi-continuous transition in their refractive index from the top (close to the refractive index of air) to the bottom (close to the refractive index of the substrate material). This gradient refractive index can effectively reduce the Fresnel reflection at the traditional “air-substrate” interface, thus ultimately achieving a surface anti-reflective effect.

**Figure 7:**
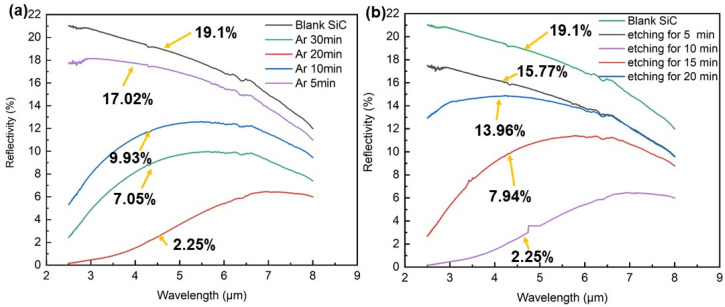
(**a**) Spectral reflectance of microcone structure with different Ar plasma treatment durations. (**b**) Spectral reflectance of microcone structure with different RIE etching times. The microcone structure formed by treating the PI layer with Ar for 20 min and etching SiC for 10 min has the lowest reflectivity of 2.25%.

To verify the anti-reflection performance of the SiC microcones, we conducted reflectivity measurements on the fabricated SiC microcone substrates. Initially, nanofibers formed by O_2_ plasma bombardment for 20 min and Ar plasma bombardment for varying durations (5, 10, 20, and 30 min) were employed as masks to etch SiC microcones with a fixed etching time of 10 min. The reflectivity of blank SiC wafers and the four types of prepared SiC substrates was measured three times, and the average values were calculated. The scanning wavenumber range was set from 4000 cm^−1^ to 400 cm^−1^ (corresponding to wavelengths of 2.5 μm–25 μm) with a resolution of 4 cm^−1^. The reflectivity test results for the obtained structures are shown in Figure 7a. As observed in the figure, with increasing Ar plasma treatment time, the average reflectivity of SiC in the mid-infrared band decreased to 17.02%, 9.93%, 7.05%, and 2.25%, respectively, compared to 19.1% for the blank SiC wafer. This reduction in reflectivity is attributed to the increased bottom diameter of the nanofibers due to prolonged argon plasma treatment, which in turn enlarged the base diameter of the microcones and enhanced their packing density for the same etching duration. The densely arranged microcone structure effectively minimizes direct light reflection. On one hand, the closely packed microcones and enlarged base diameters form a gradient refractive index layer, resulting in a smoother transition of the refractive index from air to the substrate. On the other hand, this configuration reduces Fresnel reflection at the interface, thereby significantly lowering the overall reflectivity. Notably, the structure fabricated using the nanofiber mask subjected to 30 min of Ar bombardment exhibited higher reflectivity than that formed with the 20 min Ar-treated mask. This phenomenon may be attributed to the greater height of microcones produced by the 30 min treatment, which potentially induces light-trapping effects within the nanostructured array. Thus, nanofibers formed by 20 min of O_2_ plasma treatment and 20 min of Ar plasma treatment were used as masks to fabricate SiC microcones via RIE etching for different durations (5, 10, 15, and 20 min).

The reflectivity of these four structures was measured, as shown in Figure 7b. The results demonstrate that as the etching time increased, the average reflectivity of SiC gradually decreased to 15.77%, 2.25%, 7.94%, and 13.96%, owing to the increased height of the microcones. When the etching duration was 5 min, the reflectivity was 15.77%. The reflectivity decreased with increasing etching time. However, the sample etched for 20 min exhibited a significantly higher reflectivity (13.96%) than the others, approaching that of the blank substrate. This is because excessive etching truncated the microcone tips, transforming their geometry from sharp to flat or passivated. The passivated microcones reflect light more efficiently than sharp microcones, weakening the scattering effects and electric field localization, thereby increasing the amount of light reflected. Based on these results, the optimal RIE etching time was determined to be 10 min.

By comparing the dimensions of the prepared SiC microcone structures with the optimal values obtained from the previous simulation calculations (T = 800 nm, H = 1.3 μm, and d/T = 1), it is found that the height of the prepared SiC microcone structures is consistent with the calculated value. Although the optimal period in the simulation design is 800 nm, considering that in the anisotropic etching of SiC, high-aspect-ratio structures tend to cause premature peeling of the mask, it is necessary to reduce the period to maintain structural integrity. Therefore, the period of the experimentally fabricated structure is smaller, approximately 500 nm. However, for the target mid-infrared band, both satisfy the subwavelength condition and can still excite the effective medium effect. Regarding the comparison of the filling factor, it can be concluded from Table 2 that the filling factor of the microcones with the lowest reflectivity is approximately 1, which is consistent with the aforementioned simulation calculation results. Thus, our experimental fabrication results are basically consistent with the simulation calculations. In addition, we compared the SiC microcone structures prepared by us with those in other related works, and the results of this comparison are shown in Table 3. It can be concluded that most studies on anti-reflective surfaces have focused on the visible light band, whereas the SiC microcone structures proposed in this work have achieved favorable results in the infrared band. Ultimately, the achieved reflectivity of 2.25% for the SiC anti-reflective surfaces demonstrates exceptional performance, paving the way for advanced applications in optical technologies.

## 4. Conclusions

In summary, this study successfully fabricated a gradient-refractive-index SiC microcone structure with excellent anti-reflection properties. Using nanofibers formed by sequential O_2_ and Ar plasma bombardment of PI photoresist as etching masks, we systematically investigated the morphological evolution of nanofibers under varying Ar plasma treatment durations. Because the nanofiber dimensions directly determine the resultant microcone geometry, this mask-enabled approach allows for precise control over the microcone diameter. Subsequent modulation of RIE duration further enables tunable microcone heights. The optimal SiC microcone structure (with a period of ~500 nm, height of ~1.3 μm, and fill factor of ~1) fabricated using nanofiber masks prepared with 20-min Ar plasma treatment and 10-min RIE etching achieved a remarkable reflectivity reduction of 2.25%. This gradient-index microcone architecture demonstrates outstanding broadband anti-reflection performance, offering a novel solution for enhancing optical system performance. In the future, we will continue to investigate the long-term environmental stability (such as oxidation resistance and mechanical wear resistance) of this infrared anti-reflective structure to verify its durability for practical applications. Moreover, we will explore scalable fabrication techniques for SiC infrared anti-reflective structures and integrate this structure with functional devices (such as thermal cameras, photodetectors, and remote sensing imaging equipment) to investigate its performance improvement in practical systems.

## Figures and Tables

**Table 1:** Performance comparison of SiC microcones prepared using different etching masks.

O_2_/Ar Etching	SiC Etching	d (nm)	H (nm)	T (nm)	Microcone Morphology	Reflectivity (%)
20 min/5 min	10 min	~200	~100–300	~300–500	Irregular	17.02%
20 min/10 min	10 min	~300–500	~300–800	~500–700	Irregular	9.93%
20 min/20 min	10 min	~500	~1300	~500	Regular	2.25%
20 min/30 min	10 min	~600	~1800	~500–700	Regular	7.05%

**Table 2:** Performance comparison of SiC microcones prepared with different etching durations.

O_2_/Ar Etching	SiC Etching	d (nm)	H (nm)	T (nm)	Microcone Morphology	Reflectivity (%)
20 min/20 min	5 min	/	~500	/	/	15.77%
20 min/20 min	10 min	~500	~1300	~500	Regular	2.25%
20 min/20 min	15 min	~500	~1500	~500	Tip loss	7.94%
20 min/20 min	20 min	~600	~1800	~500–700	Tip loss	13.96%

**Table 3:** Comparison of the structures, process preparation, and anti-reflective performance with the literature.

Materials	Structures	Process Preparation	Wavelength	Reflection	Ref.
SiC	Nanocones	Selective laser doping enhanced Plasma etching	3–5 μm	35%	[30]
4H-SiC	Micro-honeycomb structure	Inductively coupled plasma etching	300–1000 nm	55%	[31]
4H-SiC	Porous nanoscale periodic hole array	Photon-enhanced metal-assisted chemical etching	280 nm	10%	[32]
6H-SiC	Cone-shape nanostructures	Nanopatterning and dry etching	350–800 nm	4.2%	[33]
4H-SiC	cone array	Reactive ion etching	500 nm	10%	[34]
SiC	stochastic nanostructures	Aluminum deposition and reactive ion etching	390–784 nm	0.05%	[35]
6H-SiC	Nanocone	Self-assembled nanopatterned Reactive ion etching	390–784 nm	1.9%	[36]
SiC/MgF_2_	Double-layer anti-reflection coating	RF magnetron sputtering	475–1020 nm	0.2–3.0%	[37]
4H-SiC	SiC microcones	Reactive ion etching	2.5–8 μm	2.25%	Our work

## Data Availability

The original contributions presented in this study are included in the article. Further inquiries can be directed to the corresponding author.
